# Contribution of the Microbial Communities Detected on an Oil Painting on Canvas to Its Biodeterioration

**DOI:** 10.1371/journal.pone.0080198

**Published:** 2013-11-27

**Authors:** María del Mar López-Miras, Inés Martín-Sánchez, África Yebra-Rodríguez, Julio Romero-Noguera, Fernando Bolívar-Galiano, Jörg Ettenauer, Katja Sterflinger, Guadalupe Piñar

**Affiliations:** 1 Department of Microbiology, Faculty of Sciences, University of Granada, Granada, Spain; 2 Department of Geology and Centro de Estudios Avanzados Ciencias de la Tierra, Faculty of Experimental Sciences, University of Jaén, Jaén, Spain; 3 Department of Painting, Faculty of Fine Arts, University of Granada, Granada, Spain; 4 Institute of Applied Microbiology, Department of Biotechnology, Vienna Institute of Bio Technology (VIBT), University of Natural Resources and Life Sciences, Vienna, Austria; California Department of Public Health, United States of America

## Abstract

In this study, we investigated the microbial community (bacteria and fungi) colonising an oil painting on canvas, which showed visible signs of biodeterioration. A combined strategy, comprising culture-dependent and -independent techniques, was selected. The results derived from the two techniques were disparate. Most of the isolated bacterial strains belonged to related species of the phylum *Firmicutes*, as *Bacillus* sp. and *Paenisporosarcina* sp., whereas the majority of the non-cultivable members of the bacterial community were shown to be related to species of the phylum *Proteobacteria*, as *Stenotrophomonas* sp. Fungal communities also showed discrepancies: the isolated fungal strains belonged to different genera of the order Eurotiales, as *Penicillium* and *Eurotium*, and the non-cultivable belonged to species of the order Pleosporales and Saccharomycetales. The cultivable microorganisms, which exhibited enzymatic activities related to the deterioration processes, were selected to evaluate their biodeteriorative potential on canvas paintings; namely *Arthrobacter* sp. as the representative bacterium and *Penicillium* sp. as the representative fungus. With this aim, a sample taken from the painting studied in this work was examined to determine the stratigraphic sequence of its cross-section. From this information, “mock paintings,” simulating the structure of the original painting, were prepared, inoculated with the selected bacterial and fungal strains, and subsequently examined by micro-Fourier Transform Infrared spectroscopy, in order to determine their potential susceptibility to microbial degradation. The FTIR-spectra revealed that neither *Arthrobacter* sp. nor *Penicillium* sp. alone, were able to induce chemical changes on the various materials used to prepare “mock paintings.” Only when inoculated together, could a synergistic effect on the FTIR-spectra be observed, in the form of a variation in band position on the spectrum.

## Introduction

Nowadays, it is a well-established fact that microorganisms play an important role in the deterioration of artifacts of our cultural heritage. Two main factors are involved in the growth of microorganisms on monuments and works of art: the chemical nature of the substratum and the environmental conditions [1]. Paintings contain a wide range of organic and inorganic compounds that may be used by many microorganisms for their growth. Firstly, the materials that constitute the painting itself, i.e. the cellulose of the canvas support material, the animal glue and gypsum used to prepare a ground layer, and the linseed oil of the paint layer, are all easily degraded [2]. Secondly, the spectrum of compounds that might provide nutrients for microorganisms is further augmented by dirt, dust and other environmental contaminants deposited on the surface of the paintings [2]. This second group includes volatile hydrocarbons released from machinery, respiration and cigarette smoke, which might condense on an inert surface [3]. Furthermore, dead or living cells accumulating on the painted surface might provide an additional source of nutrients [4]. Last, but not least, microorganisms may use organic biocides to support their growth, and so the use of these chemicals in conservation practices should be controlled [5].

To date, different analytical methods have been employed for the identification of the constituent materials of paintings, and the characterization of their degradation. Cappitelli et al. [6] investigated the efficacy of IR and Raman spectroscopy in the detection of differences between native and fungal biodegraded synthetic resins. Over the last few decades, Fourier Transform-Infrared Spectroscopy (FTIR) has become one of the most important analytical techniques applied to items of cultural heritage [7] due to its versatility and ability to provide information on both organic and inorganic materials [8]. In particular, micro-FTIR, is a non-destructive surface technique that requires no sample preparation, which has improved the potential of vibrational spectroscopy and allows the identification of mixtures of compounds [9]. Thus, the constituent materials of a sample form a specific spectral fingerprint [10]. FTIR imaging enables the visualization and identification of sampling areas of interest in order to obtain the spectral data, and so spectroscopic and spatial information may be obtained simultaneously over the selected region [11].

Concerning the biodeterioration of paintings, there are a variety of mechanisms involved in this phenomenon, such as pigmentation, degradation of compounds and hydration or penetration into the materials. The successive colonization of the paintings, the excretion of aggressive metabolic products (organic or inorganic acids) and the production of extracellular enzymes, increase the loss of material [2]. The main enzymatic activities involved in the deterioration caused by microorganisms are esterases, lipases and proteases. Lipases and esterases catalyse the hydrolysis of carboxyl ester bonds [12]. Further important enzymes related to deterioration are endo-N-acetyl-P-D-glucosaminidases (ENGases), which hydrolyse the glycosidic bond between an N-acetyl-P-D-glucosamine residue and the adjacent monosaccharide within an oligosaccharide chain. Three types have been distinguished: type I, ENGases acting on murein (which forms the bacterial cell wall), type II, ENGases acting on chitin (which is the main component of the cell walls of fungi), and type III, ENGases acting on N-glycans [13]. ENGases I and II are not cleaving any painting constituent; but rather the target of these enzymes could be bacterial and fungal cells adhering to the paint layer. Some chitinases were also described as displaying a more- or less- pronounced lysozyme activity. Due to the ability of fungi to grow at low values of water activity (aw), they can produce a large amount of exoenzymes, such as cellulases, glucanases or laccases and, therefore, the preservation of oil paintings and objects of cultural heritage is concerned with the prevention, monitoring and treatment to keep fungi below dangerous levels [14].

Only the identification of the microbial communities associated with the different materials and the understanding of the role of such communities on the biodeterioration processes enable the prevention and/or remediation of the problems related to biodecay [15]. The analysis of microbial communities colonizing cultural assets was traditionally based on classical cultivation approaches, which are useful for understanding the physiological capabilities of pure isolated strains and for the development of metabolic studies. However, nowadays it is generally accepted that culture-based techniques recover less than 1% of the total microorganisms present in environmental samples [16] [17]. Hence, the study of microbial communities based only on culture-dependent methods cannot be regarded as reliable in terms of reflecting the microbial diversity present in artworks [18]. On the other hand, culture- independent techniques provide an estimate of the sequences of DNA extracted and amplified from samples [17]. Schabereiter-Gurtner et al. [19] [20] described a basic molecular protocol for the study of microbial communities colonizing cultural assets based on the sequences of the small subunit (16S for prokaryotes and 18S for eukaryotes) ribosomal RNA (rRNA) genes. Since then, many authors have used and further developed molecular protocols for the study of microbial communities colonising art works and monuments [21] [22] [23] [24] [25]. Nevertheless, it is worth noting that the best choice is the combination of classical cultivation techniques and molecular methods for the detection of the whole (cultivable and non-cultivable) microbial community in a sample.

Therefore, in this study we investigated (by culture-dependent and –independent techniques) the total microbial diversity present on an oil painting on canvas. In addition, those cultivable microorganisms showing the highest occurrence of enzymatic activities related to the deterioration processes were selected to inoculate ‘mock paintings’ (samples of painted canvases), simulating the actual layers of the investigated painting. Finally, the mock paintings were tested for their potential susceptibility to microbial degradation using micro-FTIR spectroscopy.

## Materials and Methods

### Sampling

**Figure 1:**
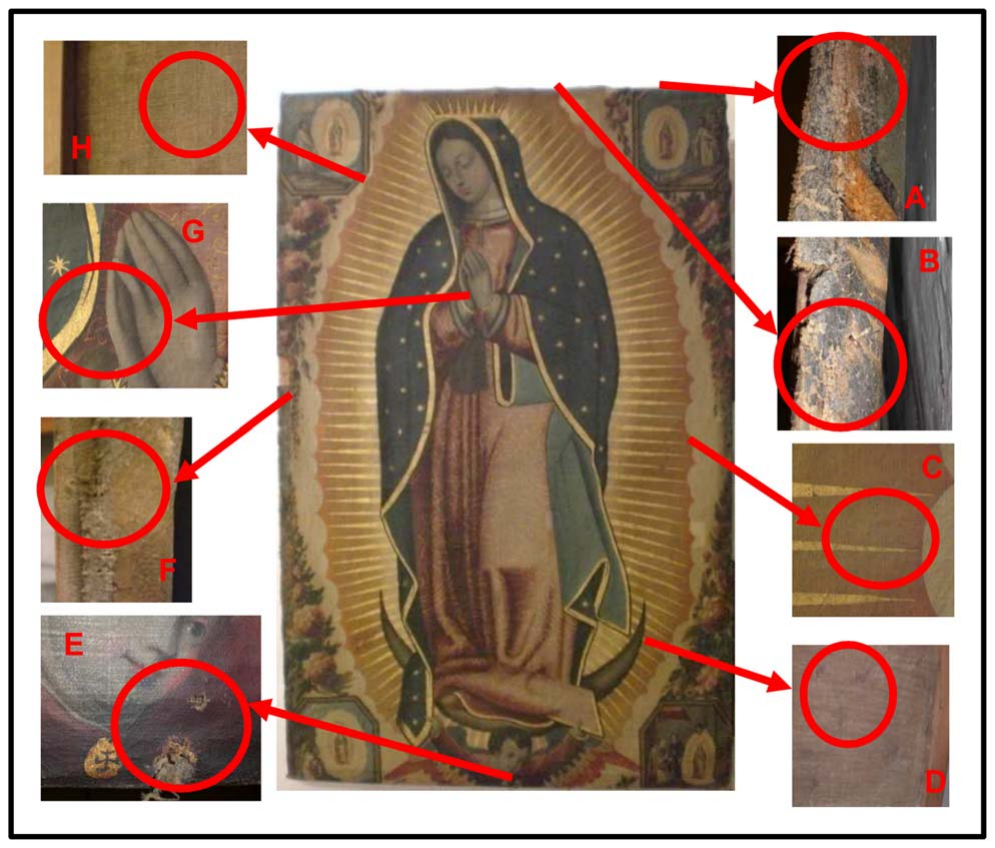
The painting “Virgen de Guadalupe” (oil on canvas) and magnification of the sampling areas. A) Sample VG1, face side of the painting; B) Sample VG2, face side; C) Samples VG7 and VG4, face side in areas showing biodeterioration signs; D) Sample VG5, reverse side in an area showing biodeterioration signs; E) Sample VG6, face side in an area showing biodeterioration signs; F) Sample VG3, face side; G) Sample VG9, face side in an area showing no signs of biodeterioration; H) Sample VG8, reverse side in an area showing no signs of biodeterioration.

Samples were taken from the oil painting on canvas “Virgen de Guadalupe”, which showed signs of biodeterioration. The painting, by an anonymous artist, was exhibited at the convent of San Antón (Granada, Spain). No specific permission was required for sampling, however the permission of the Mother Superior of the convent was sought prior to conducting the study, and the restorer Julia Ramos supervised the sampling. During a restoration campaign, two types of samples were collected to characterize the microbial community present on the painting, these being: swab samples from the surface of the painting and material scraped off the painting (Fig. 1).

Five samples were taken from the painting by a non-invasive sampling procedure [26] of rubbing a sterile and dry cotton bud, suitable for isolations in culture media (Class IIa) (Eurotubo, Deltalab, Rubí, Spain), on the surface of the painting over an area of 2 cm^2^. Three of these samples were collected from areas showing colour changes and the appearance of stains (VG5, VG6 and VG7), and the remaining two samples were collected from areas showing no visible damage, to be used as controls (VG9 and VG8). Cultivation assays were performed immediately on these samples.

Four additional samples (VG1, VG2, VG3 and VG4) were collected from areas showing signs of biodeterioration. In this case, the restorers granted permission to use invasive sampling by scraping off the biofilm on the surface of the canvas using sterile surgical scalpels (Bayha GmbH, Germany) and vials. The amount of material scraped off was ∼0.05 g from each sample. The scrapings were stored at −80°C for molecular assays.

### Cultivation strategy

Cotton swabs were inoculated directly onto agar plates containing Sabouraud-chloramphenicol Agar (Scharlau Chemie S.A., Barcelona, Spain) and Trypticase Soy Agar (TSA, Scharlau) and were incubated at 28°C, over a total period of two weeks. During this period, colonies exhibiting different morphology and appearance were transferred to new culture plates of TSA medium for bacteria and Potato Dextrose Agar (PDA, Scharlau) for fungi to obtain pure strains. All purified strains were stored in 70% glycerol at −80°C for conservation.

### DNA extraction protocols

Genomic DNA from pure bacterial strains was extracted following the procedure described by Ausubel et al. [27], which relies on a chemical disruption of cells, the removal of cell wall debris, polysaccharides and proteins by precipitation with CTAB, and the recovery of the DNA by ethanol precipitation. DNA extraction of pure fungal strains was performed according to the procedure described by Sert and Sterflinger [28], which relies on chemical and mechanical disruption of cells and the recovery of DNA by ethanol precipitation. The genotyping of pure bacterial and fungal isolated strains by Random Amplified Polymorphic DNA (RAPD) analysis was performed as described by López-Miras et al. [29].

Total DNA from the canvas material was extracted directly from the scraping of the painting (samples VG1, VG2, VG3 and VG4), according to the protocol described by Schabereiter-Gurtner et al. [19], which relies on the combination of chemical and mechanical disruption of cells and a further DNA purification by means of the QIAamp Viral RNA Mini Kit (QIAGEN GmbH, Hilden, Germany), with the modifications added by López-Miras et al. [29].

### PCR analyses using DNA extracted from canvas material

PCR reactions were executed in an MJ Research PTC-200 Peltier Thermal Cycler. For PCR reactions, 2×PCR-MasterMix (Promega, Mannheim, Germany) [50 units/ml of TaqDNA Polymerase in a supplied reaction buffer (pH 8.5), 400 µM dATP, 400 µMdGTP,400 µM dCTP, 400 µMdTTP, 3 mMMgCl_2_] was diluted to 1×, and 50 pmol/µl of each primer (VBC-Biotech, Vienna, Austria) and 2.5 µl of DNA template was added to the reaction volume. PCR reactions were carried out in 25 µl.

For the amplification of bacterial 16S rDNA fragments, primer pair 341f/985r was used under the following thermocycling conditions: five minutes denaturation at 95°C, followed by 30 cycles consisting of one minute denaturation at 95°C, one minute primer annealing at 55°C and one minute primer extension at 72°C, followed by a final extension step of five minutes at 72°C. For genetic fingerprints, a semi-nested PCR was performed in a total volume of 2×50 µl to which 2×2.5 µl template were added, using the primers 341f-GC and 518r [30] and employing the same PCR conditions as Ettenauer et al. [31].

For the amplification of fungal ITS regions, fragments of 450–600 bp, corresponding to the ITS1 and the ITS2 regions, and the 5.8S rRNA gene situated between them, were amplified with the primer pairs ITS1 forward and ITS4 reverse [32]. The thermocycling program was as follows: five minutes denaturation at 95°C, followed by 35 cycles of one minute denaturation at 95°C, one minute annealing at 55°C, and one minute extension at 72°C. Five minutes at 72°C were performed as a final extension step. For genetic fingerprints, the obtained PCR products were further amplified in a nested PCR using the ITS1 primer with an attached GC clamp on its 5′end and the reverse primer ITS2 [24]. The cycling scheme was the same as that described by Michaelsen et al. [24].

### DGGE analyses

DGGE analyses of bacterial and fungal communities were performed as described by López-Miras et al. [29]. A linear chemical gradient ranging from 30 to 55% (100% denaturant containing 7 M urea and 40% v/v formamide) was used for the screening of bacterial strains, and a gradient ranging from 20 to 55% was used for the screening of fungal communities.

### Creation of bacterial and fungal clone libraries, DGGE-screening and Sequencing analyses

Clone libraries of bacterial 16S rDNA or fungal ITS1 region fragments, were created as described by Piñar et al. [33]. Clones were screened by DGGE as described by Schabereiter-Gurtner et al. [19]. The clones displaying different DGGE-fingerprints were selected for sequencing.

For sequencing of clone inserts, 100 µl PCR products were generated with primers SP6 and T7, purified using the QIAquick PCR Purification Kit (Qiagen, Hilden, Germany) and sequenced as described by Ettenauer et al. [34]. Comparative sequence analyses were performed using the BLAST search program [35]. The sequences derived from bacterial and fungal strains and from cloned inserts have been deposited at the EMBL database under the accession numbers listed in Table 1 (bacterial and fungal clones), Table 2 (bacterial strains) and Table 3 (fungal strains).

**Table 1:** Phylogenetic affinities of partial 16S rRNA coding sequences and phylogenetic affinities of the fungal ITS1 coding region sequences detected in sample VG4 from the oil painting on canvas “Virgen de Guadalupe”.

*Clone*	*Phylum*	*Length*	*Closest identified phylogenetic relatives EMBL accession numbers]*	*Similarity (%)*	*Accession numbers of the sequences submitted to GenBank (ID:BA123456)*
**CVG B1**	Proteobacteria	564 bp	**Gammaproteobacteria** [GU991854.1; FJ976542.1]	92%	JN867741
**CVG B2**	Proteobacteria	610 bp	***Aeromonas*** ** sp**. [GQ401237.1; GQ205109.1; GQ161962.1; FJ544391.1; EU082831.1]	99%	JN867742
**CVG B18**	Actinobacteria	560 bp	***Microbacterium*** **sp**. [GQ505273.1; FJ805433.1; FJ405359.1; EF612295.1; EF204432.1; DQ227343.1; AF306542.1; AJ391205.1]	99%	JN867743
**CVG B26**	Proteobacteria	610 bp	**Gammaproteobacteria** [GU929212.1; GQ451698.1; EF446895.1]	99%	JN867744
**CVG B27**	Proteobacteria	611 bp	***Stenotrophomonas*** ** sp**. [GQ416214.1; FJ772079.1]	99%	JN867745
**CVG B33**	Actinobacteria	592 bp	***Arthrobacter*** **sp**. [FJ773996.1; FJ378036.1; EU862291.1; EF612321.1; DQ310475.1]	99%	JN867746
**CVG B34**	Firmicutes	611 bp	**Uncultured bacterium** [AJ576421.1; AJ576347.1]	99%	JN867747
**CVG B38**	Proteobacteria	609 bp	***Pseudoalteromonas*** ** sp**. [FJ662875.1; GQ885144.1; AB257325.2; FJ595988.1; EU239912.1; AB362304.1; NR_025139.1]	99%	JN867748
**CVG B40**	Firmicutes	579 bp	**Firmicutes** [HM217969.1; HM111665.1; GQ249580.1]	98%	JN867749
**CVG B44**	Proteobacteria	610 bp	**Uncultured Betaproteobacteria** [EU193046.1; EU790456.1; AY322153.1]	99%	JN867750
**CVG H67**	Ascomycota	125 pb	***Candida cellae*** strain UAF-93 [GQ149495.1]	98%	—
**CVG H68**	Ascomycota	123 pb	***Pichia pastoris*** GS115 [FN392325.1]	100%	—
**CVG H78**	Ascomycota	205 pb	***Alternaria*** ** sp**. [FJ766500.1; FJ717737.1]	100%	JN867751

**Table 2:** Molecular grouping according to RAPD analysis and identification of the bacterial strains isolated from the oil painting on canvas “Virgen de Guadalupe”.

*Representative strains from RAPD Group*	*RAPD group*	*Phylum*	*Closest related type strain on basis of 16S rRNA gene sequence*	*Similarity (%)*	*Accession numbers of the sequences submitted to GenBank (ID:BA123456)*
VG5B2	1	Actinobacteria	***Brevibacterium*** ** sp**. [DQ361016.1; DQ347560.1].	99%	JN880416
VG6B1	2	Firmicutes	***Bacillus*** ** sp**. ITP23 partial 16S rRNA gene, strain ITP23 [FR667180.1].	99%	JN867727
VG6B2; VG6B3	3	Firmicutes	***Bacillus*** **sp**. 19495 and 19493 [AJ315063.1; AJ315061.1] isolated from biodeteriorated mural paintings in the Servilia tomb (Necropolis of Carmona, Seville, Spain).	99%	JN867728
VG6B4	4	Firmicutes	***Bacillus*** **sp**. [FN870069.1; HM037905.1; HM027879.1; AB360809.1; AB298784.1; GU323365.1; GQ472195.1; GQ203617.1].	99%	JN867729
VG7B1	5	Firmicutes	***Bacillus*** ** sp**. [GU586306.1; EU810844.1].	100%	JN867730
VG7B2	6	Firmicutes	***Virgibacillus*** ** sp**. NS3012 16S ribosomal RNA gene, partial sequence [GQ889491.1].	99%	JN867731
VG7B3	7	Actinobacteria	***Arthrobacter*** ** sp**. [GU574116.1; AJ315071.1; GU323375.1].	100%	JN867725
VG7B4	8	Firmicutes	***Paucisalibacillus globulus*** partial 16S rRNA gene, type strain B22T [AM114102.1].	99%	JN867732
VG7B5	9	Actinobacteria	***Arthrobacter*** ** sp**. [GQ406812.1; FN377736.1; EU851058.1].	99%	JN867726
VG7B6	10	Firmicutes	***Paenisporosarcina*** ** sp**. [MG-2010-D29]	100%	JN867733

**Table 3:** Molecular grouping according to RAPD analysis and identification of the fungal strains isolated from the oil painting on canvas “Virgen de Guadalupe”.

*Representative strains from RAPD Group*	*RAPD group*	*Phylum*	*Closest related type strain*	*Similarity (%)*	*Accession numbers of the sequences submitted to GenBank (ID:BA123456)*
VG7H3; VG5H1	1	Ascomycota	***Penicillium*** ** sp**. isolate Y2–13 [GU134897.1].	96%	JN867738
VG6H1	2	Ascomycota	***Emericella*** ** sp**. NRRL 212 [EF652435.1].	100%	JN867734
VG6H2	3	Zygomycota	***Mucor*** ** sp**. [AY213659.1; DQ118996.1]	100%	JN867735
VG7H1	4	Ascomycota	***Penicillium*** ** sp**. [GQ121159.1].	100%	JN867736
VG7H2	5	Ascomycota	***Penicillium*** ** sp**. [GQ121159.1].	99%	JN867737
VG7H4	6	Ascomycota	***Ulocladium septosporum*** [FJ266489.1].	99%	JN867739
VG7H5	7	Ascomycota	***Eurotium*** ** sp**. [HM116370.1; EF652084.1; EF652078.1; EF652072.1; EF652070.1].	100%	JN867740

### Enzymatic characterization

The enzymatic characterization of the isolated bacterial and fungal strains was performed by using the API ZYM® system (bioMérieux, France) following the protocol of the manufacturer. This system allows the detection of the activity of 19 enzymes: alkaline and acid phosphatases, esterase (C4), esterase lipase (C8), lipase (C14), leucine, valine and cystine aminopeptidases, trypsin, α-chymotrypsin, naphthol-AS-BI-phosphohydrolase, α-galactosidase, β-galactosidase, β-glucuronidase, α-glucosidase, β-glucosidase, β-glucosaminidase, α-mannosidase, and α-fucosidase.

### Stratigraphic analyses

The stratigraphic sequence of three cross-sections of the investigated painting was identified using different techniques such as FTIR or ESEM/EDX. The cross-sections were made taking care to retain the original paint layer structure. The analyses were performed by “Laboratorio de análisis para la restauración y la conservación de obras de arte” (Villanueva de la Cañada, Madrid, Spain).

### Preparation and inoculation of “mock paintings”

Based on the stratigraphic analyses mentioned above, ‘mock paintings’ reproducing real layers of the investigated oil painting were prepared. A linen canvas was sized with animal glue (rabbit glue) in water (70 g/l) and the ground was prepared with chalk, Zinc white and animal glue. On the smoothed-out ground layer, two paint films were laid: the first composed of Sienna, which is a hydrated ferric oxide with clay minerals in linseed oil, and the second of Zinc white in linseed oil. Finally, the natural resin shellac (in solution in ethanol) was used as a varnish.

Thereafter, ‘mock paintings’ were inoculated with 20 µl of three different types of inoculum: conidia suspension containing 1×10^6^ conidia/ml of *Penicillium* sp. (7H1), bacterial suspension containing 1×10^7^ cells/ml of *Arthrobacter* sp. (7B5), and the mixture of 10 µl of bacterial suspension containing 1×10^7^ cells/ml of *Arthrobacter* sp. (7B5) and 10 µl of conidia suspensions containing 1×10^6^ conidia/ml of *Penicillium* sp. (7H1). After inoculation, each sample was placed in the center of a Petri dish and incubated for 30 days at 28°C, 75% relative humidity.

### Micro-FTIR analyses

Four different samples, sample A (‘mock painting’ inoculated with *Penicillium* sp.), sample B (‘mock painting’ inoculated with *Arthrobacter* sp.), sample C (‘mock painting’ inoculated with a mixture of *Penicillium* sp. and *Arthrobacter* sp.) and sample D (control, non- inoculated ‘mock painting’) were studied using a micro-FTIR microscope.

FTIR analyses were carried out in a JASCO FT/IR-6300 type A spectrophotometer, equipped with an IRT-7000 microscope. The FTIR absorbance measurement was performed over a range of wave-numbers between 800 and 4000 cm^−1^. The spectra were recorded at 4 cm^−1^ resolution mode with a spot of 250 µm x 250 µm, and with 500 scans for each spectrum.

Nine spectra of each sample area were collected and summed, to reduce random noise and enhance absorbance peaks [36]. The resulting spectra were compared with those of references found in other publications or deposited on databases (Database of ATR-IR spectra of materials related to paints and coatings: http://tera.chem.ut.ee/IR_spectra/index.php?option=com_content&view=article&id=94&Itemid=60) to identify the compounds present in the samples.

## Results

### Microbial diversity present on the painting

**Figure 2:**
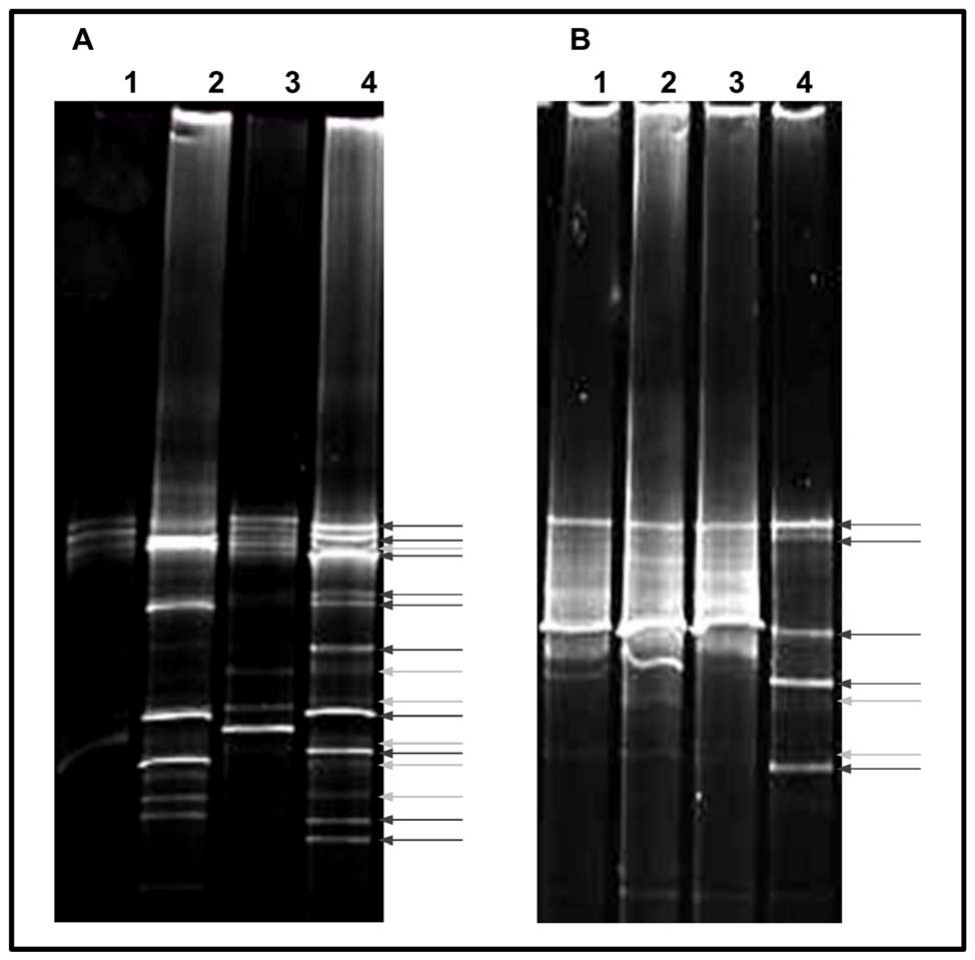
DGGE-profiles derived from total DNA directly extracted from the painting scrapes. Dark grey arrows: dominant bands. Light grey arrows: faint bands. A) DGGE-profiles of the bacterial communities. Lane 1: sample VG1; lane 2: sample VG2; lane 3: sample VG3; lane 4: sample VG4. B) DGGE-profiles of the fungal communities. Lanes as for Fig. 2A.

**Figure 3:**
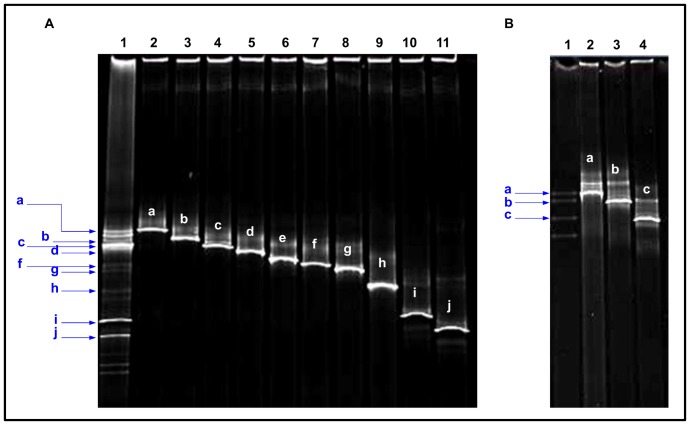
DGGE-profiles of clones containing bacterial 16S rDNA fragments (A) or the fungal ITS1 region (B), obtained from sample VG4 and producing PCR products with different motility behaviour matching the DGGE bands of the original profile. A) Bacterial clones. Lane 1: bacterial community fingerprint of sample VG4; lane 2: CVGB1; lane 3: CVGB2; lane 4: CVGB18; lane 5: CVGB26; lane 6: CVGB27; lane 7: CVGB33; lane 8: CVGB34; line 9: CVGB38; line 10: CVGB40; line 11: CVGB44. B) Fungal clones. Lane 1: fungal community fingerprint of sample VG4; lane 2: CVGH67; lane 3: CVGH68; lane 4: CVGH78.

In order to get an insight into the total microbial diversity present on the investigated painting, molecular analyses, including direct DNA extraction from sample material, amplification by using bacterial- and fungal-specific primers and fingerprinting techniques, were performed. DGGE-profiles derived from total DNA extracted from samples VG1, VG2, VG3, and VG4 revealed complex microbial communities (Figure 2A and 2B). To obtain a phylogenetic identification of the non-cultivable members, one clone library containing bacterial 16S rDNA fragments and another containing the fungal ITS1 region were generated from sample VG4, which was selected as its DGGE-profile, displayed the highest diversity. The resulting clones were screened on DGGE and inserts of clones producing PCR products which showed different motility behaviour, were selected for sequencing. Ten different clones providing sequence information of 450–611 bp of the 16S rDNA (Fig. 3A) and three clones providing sequence information between 123 and 205 bp of the fungal ITS1 region (Fig. 3B) were sequenced and compared with sequences from the EMBL database (Table 1). Results showed that the bacterial clones affiliated with species of different genera within three phyla: *Proteobacteria*, *Actinobacteria* and *Firmicutes*. We could identify species of the genera *Stenotrophomonas*, *Aeromonas*, *Pseudoalteromonas* and three unidentified members, belonging to the phylum *Proteobacteria*, species of the genera *Microbacterium* and *Arthrobacter* belonging to the phylum *Actinobacteria*, and two unidentified members of the phylum *Firmicutes*. Concerning the non-cultivable fraction of the fungal community, results showed that the fungal clones affiliated with different genera within the phylum *Ascomycota*: *Candida* sp. and *Pichia* sp., belonging to the order Saccharomycetales, and *Alternaria* sp. belonging to the order Pleosporales.

### Cultivation analyses

**Figure 4:**
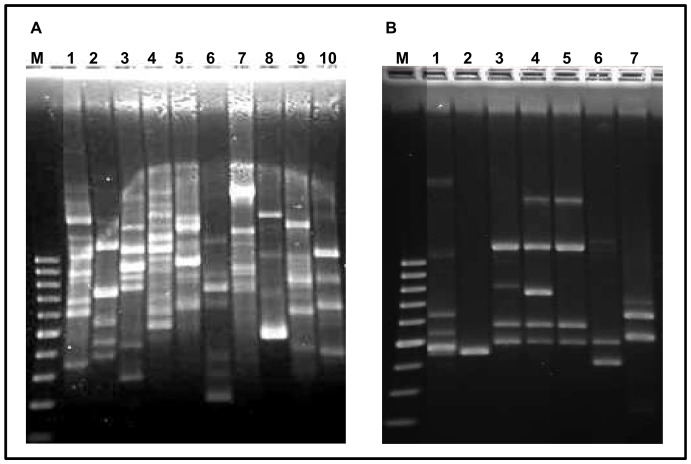
RAPD-profiles derived from one representative bacterial and fungal strain of each RAPD group. A) Bacterial strains. Lane M: 100 bp ladder; lane 1: strain VG5B1; lane 2: strain VG6B1; lane 3: strain VG6B2; lane 4: strain VG6B4; lane 5: strain VG7B1; lane 6: strain VG7B2; lane 7: strain VG7B3; lane 8: strain VG7B4; lane 9: strain VG7B6; lane 10: strain VG7B6. B) Fungal strains. Lane M: 100 bp ladder; lane 1: strain VG6H1; lane 2: strain VG6H2; lane 3: strain VG7H1; lane 4: strain VG7H2; lane 5: strain VG7H3; lane 6: strain VG7H4; lane 7: strain VG7H5.

Swab samples collected from areas showing visible deterioration signs tested positive for cultivable fungi and bacteria. A total of 11 bacterial and 8 fungal strains, differing in morphology and appearance, were isolated. In contrast, control samples collected from areas showing no visible damage yielded negative results and neither bacterial nor fungal specimens could be isolated from samples VG8 and VG9. Bacterial and fungal strains were subjected to RAPD-PCR analyses (Fig. 4) prior to sequencing. The obtained RAPD profiles allowed the clustering of bacterial strains into ten different groups (Fig. 4A) and fungal strains into seven groups (Fig. 4B). One member of each RAPD cluster was selected for sequencing and identification (Tables 2 and 3 for bacterial and fungal sequences, respectively). Similarity values ranged from 99–100% for bacteria and from 96–100% for fungi. All sequences were affiliated with cultivated bacterial or fungal strains.

Concerning the cultivable fraction of the microbial community, results derived from sequence analyses showed that bacterial strains affiliated with species of four genera (*Bacillus*, *Paenisporosarcina*, *Paucisalibacillus* and *Virgibacillus*) belonging to the phylum *Firmicutes* and with species of two genera (*Brevibacterium* and *Arthrobacter*) of the phylum *Actinobacteria*. Concerning fungi, results showed that fungal strains were affiliated with species of two different phyla: *Ascomycota* and *Zygomycota*. Within the phylum *Ascomycota*, we found three different genera belonging to the order Eurotiales (*Penicillium*, *Eurotium* and *Emericella*), and one genus of the order Pleosporales (*Ulocladium*). Within the phylum *Zygomycota*, we could only find one isolate of the genus *Mucor*.

### Enzymatic characterization

The isolated bacteria and fungi were subjected to enzymatic characterization by using the API ZYM® system (bioMérieux, France). Results derived from the enzymatic tests are presented in Table 4 and 5. The most widespread enzymatic activities related to the biodeterioration of the painting among bacterial isolates were esterase and esterase lipase; among fungi, was esterase and most of the isolates share the activity N-acetyl-β-glucosaminidase. All bacterial and fungal strains showed to be positive to naphthol-AS-BIphosphohydrolase and additionally, all fungi showed phosphatase acid activity, as shown in the tables 4 and 5.

**Table 4:** Enzymatic characterization of the cultivable bacteria. +: detectable enzymatic activity. -: non-detectable enzymatic activity.

*ENZYMATIC ACTIVITY*	*BACTERIAL ISOLATES*										
	*Brevibacterium* sp. 5B1	*Bacillus sp.*					*Virgibacillus* sp. 7B2	*Paucisalibacillus globulus* 7B4	*Paenisporosarcina* sp. 7B6	*Arthrobacter* sp.	
		6B1	6B2	6B3	6B4	7B1				7B3	7B5
**Phosphatase alkaline**	**-**	**-**	**-**	**-**	**-**	**-**	**-**	**+**	**+**	**-**	**-**
**Esterase (C4)**	**+**	**+**	**+**	**+**	**-**	**-**	**+**	**+**	**-**	**+**	**+**
**Esterase Lipase (C8)**	**+**	**-**	**-**	**-**	**+**	**+**	**+**	**+**	**-**	**-**	**-**
**Lipase (C14)**	**-**	**-**	**-**	**-**	**-**	**+**	**-**	**-**	**-**	**-**	**-**
**Leucine aminopeptidase**	**-**	**-**	**-**	**-**	**+**	**-**	**-**	**-**	**-**	**-**	**-**
**Valine aminopeptidase**	**-**	**-**	**-**	**-**	**-**	**-**	**-**	**-**	**-**	**-**	**-**
**Cystine aminopeptidase**	**-**	**-**	**-**	**-**	**-**	**-**	**-**	**-**	**-**	**-**	**-**
**Trypsin**	**-**	**-**	**-**	**-**	**-**	**-**	**-**	**-**	**-**	**-**	**-**
**α-chymotrypsin**	**-**	**-**	**-**	**-**	**-**	**-**	**-**	**-**	**-**	**-**	**-**
**Phosphatase acid**	**+**	**-**		**-**	**-**	**-**	**-**	**+**	**-**	**-**	**+**
**Naphthol-AS-BI-phosphohydrolase**	**+**	**+**	**+**	**+**	**+**	**+**	**+**	**+**	**+**	**+**	**+**
**α-galactosidase**	**-**	**-**	**-**	**-**	**-**	**-**	**-**	**-**	**-**	**-**	**+**
**β-galactosidase**	**+**	**-**	**-**	**-**	**-**	**-**	**-**	**-**	**-**	**-**	**+**
**β-glucuronidase**	**-**	**-**	**+**	**+**	**-**	**-**	**-**	**-**	**-**	**-**	**+**
**α-glucosidase**	**+**	**-**	**-**	**-**	**+**	**-**	**-**	**+**	**-**	**-**	**+**
**β-glucosidase**	**-**	**-**	**-**	**-**	**-**	**-**	**-**	**-**	**-**	**-**	**-**
**N-acetyl-β-glucosaminidase**	**-**	**-**	**-**	**-**	**-**	**-**	**-**	**-**	**-**	**-**	**-**
**α-mannosidase**	**-**	**-**	**-**	**-**	**-**	**-**	**-**	**-**	**-**	**-**	**-**
**α-fucosidase**	**-**	**-**	**-**	**-**	**-**	**-**	**-**	**-**	**-**	**-**	**-**

**Table 5:** Enzymatic characterization of the cultivable fungi. +: detectable enzymatic activity. -: non-detectable enzymatic activity.

*ENZYMATIC ACTIVITY*	*FUNGAL ISOLATES*					
	*Emericella* sp. 6H1	*Mucor racemosus* 6H2	*Ulocladium septosporum* 7H4	*Eurotium* sp. 7H5	*Penicillium* sp.	
					5H1; 7H3	7H1; 7H2
**Phosphatase alkaline**	**+**	**+**	**-**	**+**	**+**	**+**
**Esterase (C4)**	**+**	**+**	**+**	**+**	**+**	**+**
**Esterase Lipase (C8)**	**-**	**-**	**-**	**+**	**-**	**+**
**Lipase (C14)**	**-**	**-**	**-**	**-**	**-**	**-**
**Leucine aminopeptidase**	**+**	**-**	**-**	**-**	**-**	**-**
**Valine aminopeptidase**	**-**	**-**	**-**	**-**	**+**	**-**
**Cystine aminopeptidase**	**-**	**-**	**-**	**-**	**-**	**-**
**Trypsin**	**-**	**-**	**-**	**-**	**-**	**-**
**α-chymotrypsin**	**-**	**-**	**-**	**-**	**-**	**-**
**Phosphatase acid**	**+**	**+**	**+**	**+**	**+**	**+**
**Naphthol-AS-BI-phosphohydrolase**	**+**	**+**	**+**	**+**	**+**	**+**
**α-galactosidase**	**-**	**-**	**-**	**-**	**-**	**-**
**β-galactosidase**	**-**	**-**	**-**	**+**	**-**	**+**
**β-glucuronidase**	**-**	**-**	**-**	**-**	**-**	**-**
**α-glucosidase**	**-**	**-**	**-**	**-**	**-**	**-**
**β-glucosidase**	**-**	**-**	**-**	**-**	**-**	**-**
**N-acetyl-β-glucosaminidase**	**-**	**-**	**+**	**+**	**+**	**+**
**α-mannosidase**	**-**	**-**	**-**	**+**	**-**	**+**
**α-fucosidase**	**-**	**-**	**-**	**-**	**-**	**-**

*Paucisalibacillus globosus* and *Arthrobacter* sp. (Table 4) as well as *Eurotium* sp. and *Penicillium* sp. 7H1 and 7H2 (Table 5) displayed the highest number of enzymatic activities. Based on these results, one bacterial strain, *Arthrobacter* sp. (7B5) and one fungal strain, *Penicillium* sp. (7H1), were selected to assess the susceptibility to biodeterioration of the different components of the painting.

### FTIR spectroscopic analysis

**Figure 5:**
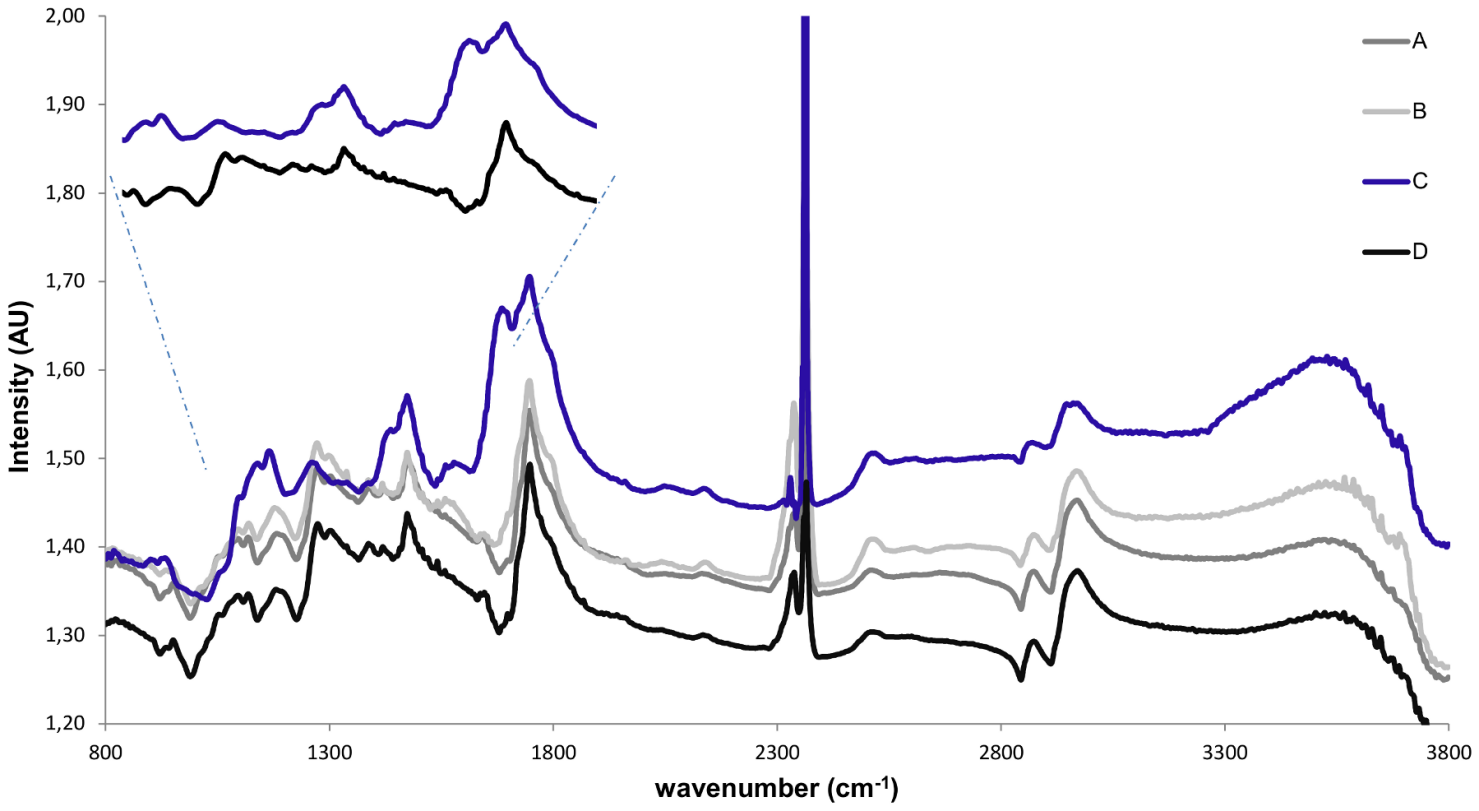
FTIR spectra of ‘mock paintings’ inoculated with *Penicillium* sp. (A), *Arthrobacter* sp. (B), a mixture of *Penicillium* sp. and *Arthrobacter* sp. (C) and the spectrum of the control (D).

Three inoculated and one non- inoculated (control) ‘mock paintings’, prepared as described in the section of methods, were studied using a micro-FTIR microscope. The IR spectra of most of the samples showed 15 main absorption bands (Fig. 5). The band assignment to chemical bonds for the vibrational IR spectrum of each sample is summarized in Table 6.

**Table 6:** Band assignments for the FTIR spectra obtained from samples A, B, C and D.υ: stretching; δ: bending; wag: wagging.

*Band position (cm^-1^)*				*Assignment*	*Painting constituent*	*References*
spectrum of sample A	spectrum of sample B	spectrum of sample C	spectrum of sample D			
1093–1098	1095–1098	absence	1093–1098	υ (O-CH_2_-C)	linseed oil	[62]
1117–1119	1119–1120	1139	1117–1119	υ (C-O) carboxilic acids	shellac	[63]
1180–1186	1175–1181	1163–1167	1179–1183	υ (C-O)	linseed oil	[62]
1271–1273	1271	1257–1267	1272–1274	υ (C-O) carboxilic acids	shellac	[63]
1299–1303	1296–1300	absence	1298–1305	C-R, C-O or C-C (wags or bends)	shellac	[58]; Database of ATR-IR spectra of materials related to paints and coatings
1387–1388	1386	absence	1386–1387	wag(CH_2_)	linseed oil	[62]
1419–1420	1418	absence	1418–1420	wag(CH_2_)-CH_2_-CO-O-	linseed oil	[62]
1474	1473–1474	1473	1473–1474	δ (CH_2_)	linseed oil	[62]
1644	1644–1645	1684–1686	1645	amide band I υ(C = O)	rabbit skin glue	Database of ATR-IR spectra of materials related to paints and coatings
1746–1748	1746–1747	1747	1747– 1748	υ (C = O)	linseed oil	[62]
2336–2337	2337	2329	2337–2338	—	shellac	[63]
2360	2363	2364	2365	—	shellac	[63]
2506–2513	2508–2518	2509–2520	2506–2519	—	chalk (CaCO_3_) mixed with linseed oil	Database of ATR-IR spectra of materials related to paints and coatings
2869–2876	2871–2874	2866–2872	2870–2876	υ (C-H) CH_2_	linseed oil	[62]
				υ (C-H) methyl	shellac	[55] [63]
2967–2971	2966–2970	2961–2962	2968– 2972	υ (C-H) CH_3_	linseed oil	[62]

No differences were detected between the spectra of samples A and B, which were single inoculated with *Penicillium* sp. and *Arthrobacter* sp. respectively, and the spectrum of the control (D). Only the IR spectra of sample C, inoculated with the mixture of *Arthrobacter* sp. and *Penicillium* sp., showed five main differences, which are summarized in Table 7.

**Table 7:** Differences between FTIR spectrum of the control (sample D) and FTIR spectrum of ‘mock painting’ inoculated with *Penicillium* sp. and *Arthrobacter* sp. (sample C) and their possible relation with the degradation of painting constituents.

*differences between band position (cm^-1^)*		*IR vibrational assignments*	*painting constituent affected*	*possible cause of the change*
FTIR spectrum of the control	FTIR spectrum of ‘mock painting’ inoculated with *Penicillium* sp. and *Arthrobacter* sp.			
presence (1093–1098 cm^−1^)	absence	υ (O-CH_2_-C)	linseed oil	esterase or esterase lipase activity
presence (1298 – 1305 cm^−1^)	absence	C-R, C-O or C-C	shellac	esterase or esterase lipase activity
presence (1386 – 1387 cm^−1^)	absence	wag (CH_2_)	linseed oil	esterase or esterase lipase activity
presence (1418–1420 cm^−1^)	absence	wag (CH_2_)-CH_2_-CO-O-	linseed oil	esterase or esterase lipase activity
presence (1645 cm^−1^)	shift to higher wavenumbers (∼1685 cm^−1^)	amide band I υ (C = O)	rabbit skin glue	modification in the state of hydration of the binder

## Discussion

### Microbial diversity revealed by culture-dependent and –independent techniques

Cultivation analyses revealed the dominance of spore-forming bacteria belonging to the phylum *Firmicutes*, principally *Bacillus*-related species, as previously found by other authors [37] [38] [39]. However, only two clones harbouring sequences of members of this phylum could be screened from the clone library. The scarcity in the detection of members of the phylum *Firmicutes* by using culture-independent methods, could be due to the low relative abundance of sequences coming from vegetative cells in the initial bacterial community, indicating that most members of this phylum isolated using culture-dependent methods are present on the painting as spores and not as vegetative cells. On the contrary, non-spore-forming bacteria belonging to the phylum *Proteobacteria* could not be isolated by cultivation methods but were shown to be dominant when total DNA was directly extracted from canvas material (sample VG4). According to Laiz et al. [22], this group may enter in a viable but non-cultivable stage (VBNC) and, therefore, may be unable to grow on the culture media used. Only species of the genus *Arthrobacter* were identified by both cultivation and molecular techniques, as previously reported [29] [40].

Concerning fungi, both culture-dependent and -independent techniques showed the dominance of members of the phylum *Ascomycota*; however, using cultivation assays, most of the fungal isolates belonged to the order Eurotiales (*Penicillium* sp., *Eurotium* sp. and *Emericella* sp.), whereas using culture-independent methods most of the fungal strains belonged to the order Saccharomycetales (*Candida* sp. and *Pichia* sp.). Only species of the order Pleosporales were detected by both cultivation and molecular techniques (*Ulocladium* sp. and *Alternaria* sp.). The impossibility of amplifying fungal DNA from members of the order Eurotiales from sample VG4 is probably due to the inefficiency of the DNA extraction method when dealing with conidia instead of mycelia. Michaelsen et al. [24] carried out a study in which the results showed that a combination of enzymatic and mechanical steps, as used in the protocol described by Schabereiter-Gurtner et al. [20], is a useful tool for the extraction of DNA from pure fungal strains existing predominantly as mycelia, but it proved to be ineffective in spore lyses. This observation may indicate that the fungi inhabiting this painting exist mainly as spores.

We cannot rule out the possibility that the discrepancies observed between the microorganisms detected with culture-dependent and –independent methods may be influenced by the different sampling methods used (rubbing or scraping off). The standardization of sampling for further culture-dependent and –independent assays is a pre-condition for microbiological analysis of environmental samples. However, when handling art works, invasive sampling (such as scraping) must be kept to the minimum, because the use of a destructive method is permissible only on fragments that cannot undergo conservation or cannot be reunited (i.e. fragments from the margins, bore dust produced by insects, biofilms, parts that will certainly be eliminated during restoration). A non-invasive sampling procedure is a requisite when working with objects of Cultural Heritage [26]. This kind of sampling can only be performed under restricted supervision and with the permission of the responsible restorers. Therefore, in this study we used cotton swabs for cultivation analyses, whenever more samples were needed.

Nevertheless, discrepancy in results when using classical cultivation versus molecular techniques is a well-known phenomenon in ecological studies [41] [42] [43] as well as in cultural heritage studies [22] [29] [31] [40], independent of the sampling procedure. For example, culture-independent techniques allowed the detection of different genera, such as the genus *Stenotrophomonas* or the genus *Microbacterium*, which could not be detected using cultivation methods, as previously observed in the study performed by Pepe et al. [18].

Culture-dependent techniques allow the detection of ∼1–5% total community, with the usual predominance of spore-forming microorganisms. The apparent abundance of spore-forming microorganisms shown by plating can be explained by the fact that they are able to grow rapidly from their spores in the culture media. Even small populations on paintings over a long period of time can produce large numbers of spores and, therefore, sampling and isolation of microorganisms from these kinds of samples may give an overestimation of the number of these microorganisms actually living on these substrates [37]. However, spore-forming microorganisms are sometimes under-screened in clone libraries, indicating possible limitations in the extraction of DNA from spores, as reported in previous works [24] [44]. Other explanations for this discrepancy can be the low relative abundance of sequences originating from vegetative cells in the initial microbial community, the use of unsuitable culture media, the non-appropriate incubation times and, last but not least, the entrance of bacteria at a viable but non-cultivable stage (VBNC), since it has already been reported that dormant microorganisms are unable to grow on standard culture media [45] [46]. As previously suggested by Pepe et al. [18], the combination of culture-dependent and -independent methods provide a better microbiological characterization. Therefore, in this study, a strategy combining culture-dependent and -independent techniques was chosen by consideration of their complementary (rather than contradictory) aspects [22] [47].

In summary, the most frequently isolated bacterial and fungal strains belonged to *Bacillus*-related species and to the genus *Penicillium*, respectively. Both have the capability to produce spores, which allow them to withstand unfavourable environmental conditions and to be quickly disseminated. Our results are in agreement with the literature data, since both genera are the most common bacteria and fungi isolated from art works and have been previously described as common inhabitants of different cultural assets [48]. Using culture-dependent methods, we were also able to isolate one member of the genus *Ulocladium*, which is frequently found on paintings and on building interiors with moisture problems [49]. In addition, one member of the genus *Eurotium*, which includes moderately xerophilic species, was identified. The genus *Alternaria* is considered to be one of the main agent of biodeterioration, owing to its ability to produce celullase that allows the degradation of the canvas of the painting [50]. Finally, yeasts are not uncommon in mural paintings or frescos, particularly on damp cellulosic materials, due to the saprophytic ability of species present in the air [51].

### Biodeteriorative potential of bacterial and fungal isolates

The pictorial surface, although susceptible to microbial colonisation, is a restrictive substrate and only those microorganisms having special metabolic (presence of hydrolysing enzymes) and physiological (production of spores, resistance to shortage of nutrients, resistance to xeric environments, etc.) capabilities may grow or survive [52] [53]. These requirements coincide with the physiological characteristics of some microorganisms detected in this study, as mentioned above. The detection of airborne bacteria and fungi which may be considered to be transient microorganisms, and not the ones involved in the deterioration of the painting, highlights the necessity to clarify their role in the biodeterioration process [20]. These airborne spores and cells may be deposited onto the painted surface in the indoor environment by gravitational settling or carried by the wind. Consequently, we decided to carry out an enzymatic characterization of the cultivable bacteria (Table 4) and fungi (Table 5) to elucidate whether indeed, these microorganisms have the metabolic and destructive potential to be responsible for the biological degradation of the investigated painting and whether they represent a real risk for its future biodeterioration.

Results showed that the naphthol-AS-BI-phosphohidrolase activity was detected in all strains, and phosphatase acid in all fungi, which are standard phosphatases. In addition, the most widespread enzymatic activities related to biodeterioration were, among bacterial isolates, esterase and esterase lipase activities, and among fungal isolates, esterase activity. Both enzymes are able to catalyse the hydrolysis of ester bonds, so these enzymes might take part in the degradation of painting constituents such as linseed oil or shellac. Linseed oil is a natural drying oil, composed of a mixture of triglycerides derived primarily from unsaturated oleic, linoleic and linolenic acids [54], which have been used extensively as a medium for painting. Shellac is a sesquiterpenic resin secreted by an insect (*Kerria lacca*, Hemiptera order) and composed mainly of polyesters of oxyacids [55]. Most fungi showed the presence of the activity of N-acetyl-β-glucosaminidase, which is able to hydrolyze the linkage in the polysaccharide backbone of bacterial cell wall peptidoglycan. This enzyme, together with other enzymatic activities, could convert the peptidoglycan to oxidizable monosaccharides when no nutrients are available [56]. Moreover, bacterial cell wall peptidoglycan contains nitrogen, which is essential for the synthesis of proteins. Thereby, this enzymatic characterization demonstrates that most fungi and bacteria isolated in this study displayed the necessary metabolic potential to be responsible for the biological attack observed on the painting prior to the time of sampling or may represent a risk of future biodeterioration.

### Biodeterioration process on “mock paintings”

The isolation of cultivable bacteria and fungi exclusively in the damaged areas of the painting and the absence of this community in undamaged areas may indicate the possible involvement of certain members of the community in the deterioration of the painting; probably those microorganisms detected by both techniques, such as the genus *Arthrobacter*. Moreover, *Arthrobacter* sp. is a facultatively oligotrophic genus resistant to high salt concentrations, desiccation and starvation [22]. Between the isolated fungi, *Eurotium* sp. and *Penicillium* sp. (7H1 and 7H2) showed the same enzymatic activities; however, *Penicillium* sp. was the most abundant among the isolated fungal genera. Therefore, *Penicillium* sp. (7H1) and *Arthrobacter* sp. (7B5) were selected for the assessment of the susceptibility to biodeterioration of the different material components of the painting. They were chosen because of their resistance to adverse environmental conditions and their enzymatic potential.

We were able to recover fungal cells on PDA medium from ‘mock paintings’, even after 30 days of incubation (data not shown) only when we inoculated together *Arthrobacter* sp. and *Penicillium* sp.. This suggests that the presence of bacterial cells somehow favour survival of *Penicillium* sp. on ‘mock paintings’. Our working hypothesis is that the enzyme N-acetyl-β-glucosaminidase, exhibited by this fungus, may participate in the hydrolysis of the bacterial cell wall peptidoglycan, rendering oxidizable monosaccharides capable of supporting fungal growth. Thus, dead bacterial cells adhering to the paint layer make a nutritional contribution to fungal growth, as previously suggested by Guglielminetti et al. [51]. In addition, this fungus may require a period of growth and adaptation to the substrate before starting to degrade some components of the pictorial layer.

Concerning the FTIR spectroscopic analysis, we were able to detect five main differences (Table 7) between the FTIR spectrum of the control and the FTIR spectrum of ‘mock painting’ inoculated with *Penicillium* sp. and *Arthrobacter* sp., indicating that a synergistic effect between certain microorganisms, more than the sole action of a single strain, may increase the biodeterioration process of the materials that constitute the painting. In micro-FTIR techniques, an infra-red spectrometer is linked to a microscope, so the sampling areas of interest can be visually identified in order to obtain the spectral data. Since all the intensities are relative, no direct quantitative comparison can be performed between intensities of different bands [57].

One difference was the absence of the vibration band at 1298–1305 cm^−1^, which can be assigned to C-R, C-O or C-C wags and bends [58], originated from shellac. Other differences were the absence of the vibration band at 1093–1098 cm^−1^ (assigned to O-CH_2_-C stretching vibration), the absence of the vibration band at 1386–1387 cm^−1^ (assigned to CH_2_ wagging vibration) and the absence of the vibration band at 1418–1420 cm^−1^ (assigned to (CH_2_)-CH_2_-CO-O- wagging vibration), all of them originated from linseed oil. A possible cause of these changes may be related to the activity of an esterase or an esterase lipase (hydrolases). The fact that *Penicillium* sp. displayed both enzymatic activities, as previously detected in the enzymatic characterization, may suggest the possible involvement of an esterase or an esterase lipase in the biodeterioration process of linseed oil and shellac.

Finally, the standard protein binder selected to prepare the samples was rabbit skin glue (collagen) which, since ancient times, has been used in traditional woodworking, gilding and paintings due to its high strength, viscosity and elasticity [59]. The amide I band (∼1655 cm^−1^) is due almost entirely to the C = O stretch vibrations of the peptide linkages, with some contribution of CN stretching, and this band has proven to contain significant information about the protein secondary structure [60]. The shift observed in the amide I vibration band to higher wavenumbers (∼1685 cm^−1^) with respect to the values observed for the controls (1645 cm^−1^), indicates a modification in the state of hydration of the binder [60]. Depending on the hydration energy and steric hindrance of the anions, salts may promote aggregation, unfolding, or dissociation of the protein [61].

## Conclusions

The results obtained in this study highlight firstly that microbiological and molecular analyses are complementary in evaluating the microbial community dwelling on a deteriorated oil painting on canvas. Secondly, it was necessary to distinguish between which microorganisms were involved in the observed deterioration, and which were merely ubiquitous air-borne organisms not implicated in the damage, by determining the metabolic activities displayed by isolated fungal and bacterial strains. Finally, FTIR analyses performed on ‘mock paintings’ demonstrated that the synergistic action of certain microorganisms present on the painted surface, more than the sole action of a single strain, such as *Arthrobacter* sp. and *Penicillium* sp. in this study, may be involved in the biodeterioration of the materials that constitute the painting. Nevertheless, even if the microorganisms detected using culture-dependent techniques are not directly responsible for the current damage of the painting, a change in environmental conditions could promote their growth and produce further damage, since the fungal and bacterial strains isolated in this study displayed metabolic activities with the potential to be responsible for the biological attack observed on the painting. This fact indicates the importance of keeping the painting under good preservation conditions after any cleaning or disinfection campaign.
